# Evolutionary divergence in the fungal response to fluconazole revealed by soft clustering

**DOI:** 10.1186/gb-2010-11-7-r77

**Published:** 2010-07-23

**Authors:** Dwight Kuo, Kai Tan, Guy Zinman, Timothy Ravasi, Ziv Bar-Joseph, Trey Ideker

**Affiliations:** 1 Departments of Bioengineering and Medicine, University of California San Diego, 9500 Gilman Drive, La Jolla, CA 92093, USA; 2 Departments of Internal Medicine and Biomedical Engineering, University of Iowa, 200 Hawkins Drive, Iowa City, IA 52242, USA; 3 Department of Computer Science, Carnegie Mellon University, 500 Forbes Avenue, Pittsburgh, PA 15213, USA; 4 Red Sea Laboratory of Integrative Systems Biology, Division of Chemical and Life Sciences and Engineering, Computational Bioscience Research Center, King Abdullah University of Science and Technology, Thuwal 23955-6900, Kingdom of Saudi Arabia

## Abstract

**Background:**

Fungal infections are an emerging health risk, especially those involving yeast that are resistant to antifungal agents. To understand the range of mechanisms by which yeasts can respond to anti-fungals, we compared gene expression patterns across three evolutionarily distant species - *Saccharomyces cerevisiae*, *Candida glabrata *and *Kluyveromyces lactis *- over time following fluconazole exposure.

**Results:**

Conserved and diverged expression patterns were identified using a novel soft clustering algorithm that concurrently clusters data from all species while incorporating sequence orthology. The analysis suggests complementary strategies for coping with ergosterol depletion by azoles - *Saccharomyces *imports exogenous ergosterol, *Candida *exports fluconazole, while *Kluyveromyces *does neither, leading to extreme sensitivity. In support of this hypothesis we find that only *Saccharomyces *becomes more azole resistant in ergosterol-supplemented media; that this depends on sterol importers Aus1 and Pdr11; and that transgenic expression of sterol importers in *Kluyveromyces *alleviates its drug sensitivity.

**Conclusions:**

We have compared the dynamic transcriptional responses of three diverse yeast species to fluconazole treatment using a novel clustering algorithm. This approach revealed significant divergence among regulatory programs associated with fluconazole sensitivity. In future, such approaches might be used to survey a wider range of species, drug concentrations and stimuli to reveal conserved and divergent molecular response pathways.

## Background

Mucosal and invasive mycoses are a major world health problem leading to morbidity [1,2] and a mortality rate of up to 70% in immunocompromised hosts [3]. The most common treatment for fungal infections is the family of chemical compounds known as the azoles, which interfere with formation of the cell membrane by inhibiting synthesis of ergosterol [4]. However, the use of azoles to treat a broad spectrum of fungal infections has led to widespread azole resistance [4-9], and resistance is also emerging against the limited number of secondary compounds that are currently available [10,11].

The fungal response to azoles has been most often studied in yeast [5,7,12-17], primarily through analysis of standard laboratory strains of *Candida *[12,13,18] or *Saccharomyces *[14,16,17] or their resistant clinical isolates [2,12,15,19]. Other studies have focused on cultures for which drug resistance has been artificially evolved *in-vitro *[15,18,20,21]. This work has revealed a number of resistance and response mechanisms that can be invoked to protect cells from drugs, including mutations to drug efflux pumps or their regulators [2,12,20,21], mutations to ergosterol synthesis enzymes [20], duplication of the fluconazole target Erg11 [18], and a possible role for Hsp90 [15,22].

Although these represent a wide array of mechanisms, it is likely that the full range of anti-fungal resistance pathways is even greater, for several reasons. The first relates to genetic diversity: the number of clinical isolates that have been studied to-date is relatively modest, and resistant strains produced by artificial evolution are only a few generations removed from the common laboratory strains used as starting material. The second reason relates to the environment: it is very difficult to mirror in the laboratory the range of conditions that must be experienced by yeast in the wild during the evolution of stress response pathways. Thus, an important goal moving forward is to better understand the entire pool of genotypic variation underlying fungal stress responses, particularly as they relate to antifungal agents.

Towards this goal, we performed a comparative study of the transcriptional program activated by fluconazole in three evolutionarily distinct yeasts: *Saccharomyces cerevisiae *(*Sc*), *Candida glabrata *(*Cg*), and *Kluyveromyces lactis *(*Kl*). These species were selected to provide a survey of transcriptional networks at intermediate evolutionary distance, that is, at sufficient distance to observe evolutionary change but sufficiently close to ensure significant conservation. *Sc *and *Cg *diverged approximately 100 million years ago, and both harbor evidence of an ancient whole-genome duplication event [23]. *Cg *is an established human pathogen while *Sc *has been occasionally found to cause systemic infection in immunocompromised individuals [2]. *Kl *was selected as an outgroup since its evolutionary history is clearly distinct from *Sc *(having diverged prior to whole-genome duplication [24]) but its transcriptional network is substantially closer to *Sc *than, for instance, is the network of *Candida albicans *[25]. In addition, *Sc*, *Cg*, and *Kl *share functional and phenotypic characteristics (for example, growth as haploids [26], similar codon usage [26]) that make them suitable for comparison.

Earlier efforts to profile expression across different species have been limited to the examination of matched conditions across two organisms [27-29] or curated compendia of microarrays across many conditions [24,30,31]. Such studies have previously identified transcriptional mechanisms leading to large phenotypic divergence among yeasts, often related to the whole-genome duplication event [24,30,31]. Accordingly, we reasoned that matched expression time courses of three yeasts might reveal evolutionary differences in the transcriptional stress response elicited by an anti-fungal drug.

## Results and discussion

### *Kl *is dramatically more sensitive to fluconazole than other species

For each of the three species *Sc*, *Cg*, and *Kl*, we obtained standard laboratory strains for which genome sequences were available (Materials and methods). We examined the phenotypic response of these species to a range of concentrations of fluconazole (Additional file 1: Testing Fluconazole Susceptibility), a triazole antifungal drug commonly used in the treatment and prevention of superficial and systemic fungal infections [4]. We found that *Kl *was approximately 70 times more sensitive to fluconazole than *Sc *and *Cg*, with a 50% inhibitory concentration of 0.06 μg/ml versus 4.0 μg/ml for both *Sc *and *Cg *(Figure S1 in Additional file 1). Cross-species differences in sensitivity could be due to a variety of factors, including differences in membrane permeability or drug transport, divergence in sequence or regulation of the drug target Erg11, or in any of the pathways previously linked to azole resistance.

### Comparative expression profiling of *Sc*, *Cg*, and *Kl *

**Figure 1:**
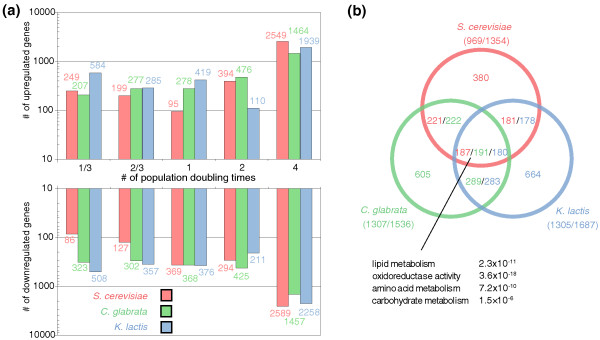
**Differentially expressed genes**. **(a) **Number of differentially expressed (up- and down-regulated) genes by species versus the number of cell doublings. **(b) **Venn diagram showing the overlap in the sets of differentially expressed genes selected in each species at a false discovery rate of *q *≤ 0.1. The number of differentially expressed genes in each region of the Venn diagram is not identical across species, since the number of genes that a species contributes to an orthologous group (that is, number of paralogs) can vary. Ratios in parentheses indicate the number of differentially expressed orthologs by the total number of differentially expressed genes (not all genes possess orthologs).

While it is possible that complementary strategies might be observed at different fluconazole dosages [20], we exposed each species to fluconazole at its 50% inhibitory concentration to facilitate direct comparison of the transcriptional response between species. We then monitored global mRNA expression levels at 1/3, 2/3, 1, 2, and 4 population doubling times (Figure 1a). We also found that sampling based on the doubling time of each species, as opposed to absolute time measurements, led to greater coherence in the expression profiles across species (Figure S2 in Additional file 1; Additional file 1: Analysis of Doubling Time Points vs. Absolute Time Points). Selected mRNA measurements were validated using quantitative RT-PCR against six genes (Figure S3 in Additional file 1). We also found significant overlap of the *Sc *differentially expressed genes with several previous microarray studies and some overlap with gene deletions conferring fluconazole sensitivity (Additional file 1: Microarray Design and Analysis).

To compare expression profiles across species, orthologous genes were defined using MultiParanoid [32]. As might be expected based on known phylogenetic distances [23], *Cg *shared more differentially expressed genes with *Sc *than with *Kl *(Figure 1b). We also found some overlap with previously published *C. albicans *microarray data, especially with the functions of the responsive genes such as those involved in ergosterol biosynthesis and oxido-reductase activity (Additional file 1: Microarray Design and Analysis).

### Soft clustering: a novel cross-species clustering algorithm

**Figure 2:**
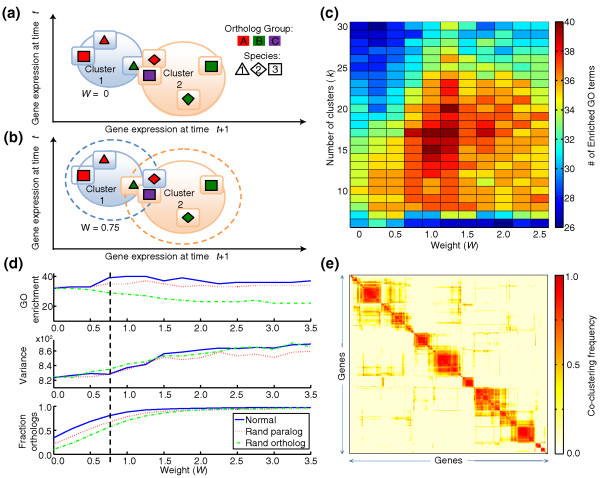
**Soft clustering method**. **(a) **Standard clustering based on expression only: two sets of orthologs are depicted (color represents orthology, shape represents species) where orthologs are split between clusters 1 and 2. For illustrative purposes, only two time points (*t *and *t *+ 1) are shown. **(b) **Soft clustering based on expression and orthology: dashed circles denote regions where orthologs will be co-clustered. Since the purple square has no orthologs in cluster 1, it remains assigned to cluster 2. **(c) **Effect of number of clusters *k *and orthology weight *W *on GO term enrichment. **(d) **The number of enriched GO terms, variance, and fraction of co-clustered orthologs for *k = *17 as a function of *W *in comparison to randomized paralogs/orthologs. Randomization was performed as described in Additional file 1: Randomizing the Orthology Mapping. **(e) **Since *k*-means is non-deterministic, to ensure robustness we performed 50 runs of the algorithm recording the fraction of times each gene pair was co-clustered (including all genes from all species). This matrix was hierarchically clustered.

Due to factors such as measurement error and ambiguity of cluster boundaries, we found that the available clustering methods led to situations in which orthologous genes with similar expression patterns could be misplaced into different clusters (Additional file 1: Constrained Clustering Algorithm). Accordingly, we developed a 'soft' clustering approach that integrates expression profiles with gene sequence orthology in a modified *k*-means model. This algorithm includes an adjustable weight that rewards ortholog co-clustering (Figures 2a, b; Materials and methods; Additional file 1: Constrained Clustering Algorithm). The term 'soft clustering' has also previously been used in other clustering methods to define cases in which a gene can belong to more than one cluster rather than any constraint used to identify clusters [12,13]. Unlike standard clustering methods, which focus solely on cluster coherence, the soft clustering method can simultaneously detect both similar and divergent behavior between orthologs. For instance, when orthologs are not co-clustered despite the addition of a reward, one can be assured that their dynamic profiles truly differ. The weight *W *and the number of clusters *k *were scanned over a range of values (Figure 2c). We selected *W = *0.75 and *k *= 17 as choices that approximately optimized the enrichment for Gene Ontology (GO) terms (Additional file 1: Constrained Clustering Algorithm; Additional file 1: Selecting Parameters for the Constrained Clustering Method).

**Figure 3:**
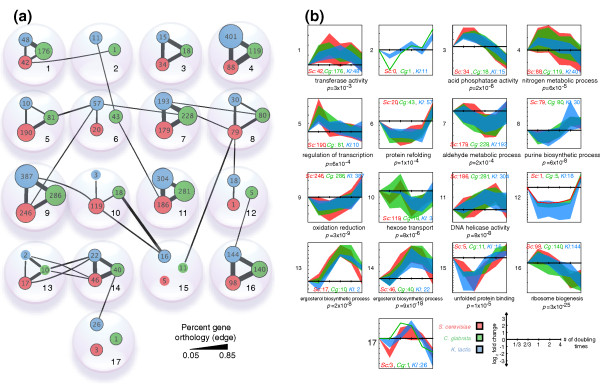
**Cluster structure and dynamics**. **(a) **Each of the 17 clusters appears as a bubble containing up to three colored nodes whose sizes represent the number of genes contributed by each species. Edge thickness denotes the percent of gene orthology shared within or between clusters, measured using the size of the intersection divided by the size of the union of the sample sets. Only significant edges (*P *< 0.01) are shown. Several clusters show conserved orthology but not dynamics (for example, cluster 10 *Sc*, *Cg *with cluster 15 *Kl*). Note that clusters were ordered to minimize orthology edge crossings. **(b) **Expression dynamics of the 17 soft clusters over time following fluconazole exposure. Separate plots for each species can be found in Additional file 1. The width of each band corresponds to ± one standard deviation about the mean. A selection of enriched GO terms are shown for different clusters; see Figure S11 in Additional file 1 for full GO enrichment results. The number of genes for each species in each cluster is also shown.

We compared our soft clustering approach to additional standard clustering methods (Figure S4a in Additional file 1). In comparison to classical *k*-means (equivalent to *W *= 0), the fraction of co-clustered orthologs increased from approximately 35% to 70%, with a negligible increase in within-cluster variance (Figure 2d). For *W *> 0.75, we saw no improvement in the number of enriched GO terms, a marked increase in total cluster variance, and little improvement in the fraction of co-clustered orthologs (Additional file 1: Constrained Clustering Algorithm). Since *k*-means is non-deterministic, to ensure robustness the results of 50 runs of the algorithm were used to populate a matrix recording the fraction of times each gene pair was co-clustered. This matrix was used as a similarity matrix for subsequent hierarchical clustering (Figure 2e; Additional file 1: Co-clustering Matrix). The resulting 17 cross-species gene expression clusters are shown in Figure 3a, b, Figure S7 in Additional file 1, and Table S1 in Additional file 2.

### Conservation of *cis*-regulatory motifs across clusters

We found that two cross-species clusters (13 and 14) were highly enriched for ergosterol biosynthetic genes (*P *≤ 10^-8^) and were coherently up-regulated in all three species - likely in response to ergosterol depletion. Both clusters were also enriched for the upstream DNA-binding motif of the sterol biosynthesis regulators Ecm22 and Upc2 [33]. Interestingly, Upc2 has also been implicated in increased fluconazole resistance in the fungal pathogen *C. albicans *[34]. Rox1 motifs were enriched in *Sc *and *Cg *but not *Kl*. A likely explanation for this divergence is that Rox1 is a repressor of hypoxia-induced genes, and *Kl *both lacks a Rox1 ortholog and the capacity for anaerobic growth.

Beyond the clusters representing ergosterol biosynthesis, we found two additional clusters (9 and 16) in which high conservation of expression patterns, sequence orthology, and *cis*-motif conservation were observed across species. Cluster 9 was regulated by the general stress-response transcription factors Msn2p and Msn4p (*q *< 10^-5^; Additional file 1: Expression Conservation of the General Stress Response) and showed GO enrichment for oxido-reductase activity (*q <*10^-8^) and carbohydrate metabolism (*q <*10^-7^). Cluster 16 was enriched for ribosomal biogenesis and assembly (*q <*10^-13^) with upstream PAC [35] and RRPE motifs previously implicated in regulating genes involved in the general stress response and ribosomal regulation (Additional file 1: Expression Conservation of the General Stress Response) [28,31,35,36].

For other clusters, conserved motifs were absent, suggesting divergence across species. This lack of motif conservation was particularly surprising for clusters 3, 4, 7, and 11, which contained large numbers of co-expressed orthologous genes. On the other hand, this finding is consistent with previous studies finding low motif conservation [24,28,30,31]. We also found no significant enrichment for binding sites of orthologous transcription factors (Tac1, Mrr1, Crz1) known to mediate fluconazole-resistance in the evolutionarily diverged pathogen *C. albicans *[37].

Despite application of the soft-clustering algorithm, some clusters nevertheless shared significant gene orthology (but not expression) with other clusters, such as clusters 10 and 15 in Figure 3a. In these cases, we also found no conserved motifs between these clusters, indicating both promoter and expression divergence among orthologs in addition to species-specific motifs (Additional file 1: Species-specific Motifs).

### Co-clustering implicates both highly conserved and divergent pathways

**Figure 4:**
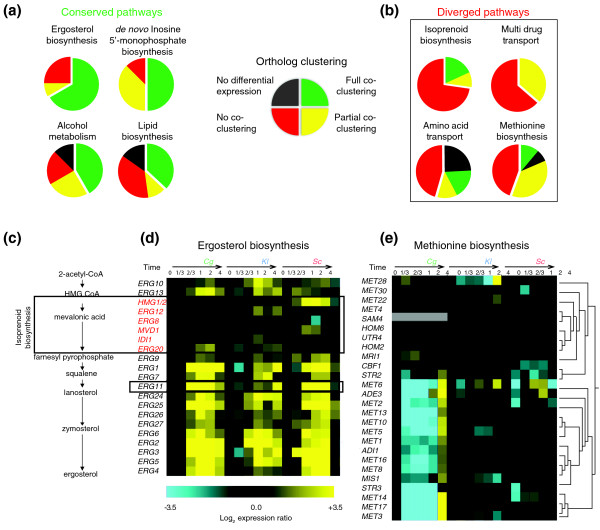
**Pathway expression conservation and divergence**. **(a) **Top conserved and **(b) **diverged pathway responses as revealed by the soft clustering approach. Each pathway is represented by a pie with four slices - green, yellow, red, and black - denoting the percentage of orthologs in that pathway for which all three species co-clustered, two species co-clustered, no two species co-clustered, and no species' orthologs were differentially expressed, respectively. Pathways were defined using GO biological process annotations. **(c) **Schematic of ergosterol biosynthesis, the most conserved pathway response. Interestingly, this pathway includes isoprenoid biosynthesis, for which the response was one of the most divergent. **(d) **mRNA expression responses of ergosterol pathway genes are shown in order of occurrence in the pathway. Expression levels of genes 3 to 8 (boxed, and red) corresponding to isoprenoid biosynthesis are strikingly divergent. The fluconazole target Erg11 is boxed. **(e) **Hierarchically clustered mRNA expression responses of methionine biosynthesis genes show extensive divergence across species. Grey expression values denote a gene for which the species lacks an ortholog.

Next, we analyzed the soft clusters to identify pathways for which the fluconazole response is either highly conserved or strikingly divergent. For this purpose, differentially expressed pathways were identified using the GO Biological Process database [38] (Materials and methods). For each pathway, we computed the number of orthologous gene groups for which: 1, all three species were in the same cluster (full co-clustering); 2, two species were in the same cluster (partial co-clustering); or 3, no two species were in the same cluster (no co-clustering). The pathways with the highest percentage of orthologs with full co-clustering are shown in Figure 4a. The pathways with the highest percentage of orthologs that do not co-cluster are shown in Figure 4b. Clustering results for all pathways are given in Table S2 in Additional file 3.

By this analysis, the most conserved pathway was ergosterol biosynthesis, which is consistent with our study of conserved motifs (above). Fluconazole directly inhibits ergosterol synthesis by targeting of Erg11, and all species appear to respond strongly to this reduction in ergosterol by up-regulating the enzymes required for its novel biosynthesis. *ERG11 *was up-regulated early in both *Sc *and *Cg *and later in *Kl*. Since *ERG11 *over-expression is one mechanism by which yeast can overcome fluconazole-induced growth inhibition [18], delays in its induction could contribute to *Kl*'s greater fluconazole sensitivity.

The first stages of ergosterol biosynthesis are carried out by a subset of enzymes of the isoprenoid pathway. While most ergosterol genes were coordinately up-regulated in all three species, the expression levels of isoprenoid biosynthesis genes were strikingly divergent (Figures 4b, d). In all eukaryotes, regulation of isoprenoid biosynthesis is known to be complex with multiple levels of feedback inhibition [39]. Thus, the extensive divergence in isoprenoid biosynthesis expression suggests that the regulation of this pathway has also diverged between species.

Extensive expression divergence was also observed in methionine biosynthesis and amino acid transport (Figure 4b). Curiously, many *Cg *methionine biosynthesis orthologs were strongly down-regulated early in the time-course (Figure 4e). This strong down-regulation was not mirrored in *Sc *and *Kl*, which displayed divergent expression responses that were not co-clustered. Interestingly, it has been previously suggested that differences in methionine biosynthesis may alter azole susceptibility in *C. neoformans *[40] and *C. albicans *[41].

### Major divergence in mRNA expression of transporters

**Figure 5:**
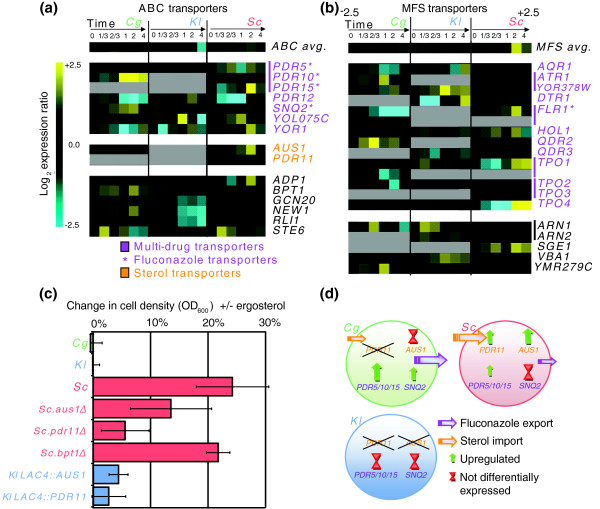
**Divergence in transporter usage**. Cross-species expression profiles of **(a) **ATP-binding cassette (ABC) and **(b) **major facilitator superfamily (MFS) transporters are shown. Grey expression values denote a gene for which the species lacks an ortholog. **(c) **Change in cell density with addition of exogenous ergosterol at the fluconazole 50% inhibitory concentration across different mutant backgrounds. *Sc.bpt1Δ *is a gene knockout unrelated to fluconazole response and is included as a control. Error bars indicate one standard deviation. **(d) **Model for differential usage of transporters among *Sc*, *Cg*, and *Kl*.

A final pathway for which we observed striking expression divergence was multi-drug transport (Figure 4b; Additional file 1: Transport). Most genes in this pathway were covered by clusters 8, 11, 16 (Figure 5a, b). Multi-drug transporters are divided into two classes: ATP-binding cassette (ABC) and major facilitator superfamily (MFS) transporters [5]. We examined the expression patterns of these transporters and found at least two types of divergent behaviors. First, the fraction of differentially expressed *Sc *MFS transporters was low compared to *Cg *and *Kl *(Fisher exact test, one-tailed *P = *0.025 and 0.020, respectively). Second, the timing of MFS gene expression differed, with *Sc *up-regulated late and *Cg *up-regulated early (Figure 5b). In *SC*, several ABC and MFS transporters have been shown to bind fluconazole as a substrate [20,42,43]. Of these, we found that the *PDR5*/*10/15 *family of ABC transporters was up-regulated in *Cg *and *Sc *but not *Kl*. Another fluconazole transporter, *SNQ2*, was up-regulated in *Cg *only.

We also found strong differences in the expression of other multi-drug transporters that have not been previously linked to fluconazole: *PDR12 *was strongly down-regulated in *Sc *and *Cg *but up-regulated in *Kl*; *ATR1 *and *YOR378W *were up-regulated in *Cg *and *Kl *but not *Sc*; *HOL1 *was up-regulated in *Sc *and *Kl *but not *Cg*. Some transporters also showed differences in expression timing (*YOR1*, *PDR12*).

Additionally, two ABC transporters, *AUS1 *and *PDR11*, which uptake sterol under anaerobic conditions [44], were up-regulated in *Sc *but were not differentially expressed in *Cg *(*Cg *does not possess a *PDR11 *ortholog). This suggests that *Sc *but not *Cg *increases sterol transport during fluconazole exposure. Intriguingly, since the direct effect of fluconazole is to inhibit sterol synthesis, increased sterol transport could be a mechanism for increased fluconazole tolerance. In support of this hypothesis, we found that the normally repressed cell wall mannoprotein DAN1, whose expression is required for sterol uptake [45], was up-regulated in *Sc *but not *Cg*. Since *Kl *lacks sterol transporters, it cannot import sterol and only grows aerobically [46,47] (Additional file 1: Analysis of Sterol Import Machinery in Fungal Genomes). As a possible explanation for this divergent behavior, we found that the promoter regions of *ScAUS1*, *ScPDR11*, and *ScDAN1 *contain binding motifs for ergosterol biosynthesis and/or sterol transport regulators Ecm22p, Rox1p and Sut1p, all of which were absent upstream of *CgAUS1 *and *CgDAN1*.

Therefore, the striking divergence in expression of fluconazole export and sterol import pathways suggests differing strategies in the azole response: following fluconazole exposure, *Sc *appears to activate sterol influx through up-regulation of *PDR11 *and *AUS1*; in contrast, *Cg *may activate fluconazole efflux through strong up-regulation of *SNQ2 *and a *PDR5*/*10/15 *ortholog (Figure 5a).

### Sterol import increases fluconazole tolerance in *Sc*, but not *Cg *or *Kl *

To investigate these hypotheses, we grew wild-type *Sc *and *Cg *along with deletion mutants *Sc.aus1Δ *and *Sc.pdr11Δ *under fluconazole treatment in the presence or absence of exogenous ergosterol (4 μg/ml). As shown in Figure 5c, we found that addition of ergosterol had no effect on growth of *Cg *but led to an increase in growth of *Sc *(*P *= 0.018). This increase was attenuated in *Sc.aus1Δ *and *Sc.pdr11Δ *(*P *= 0.033), which lack sterol import genes, but not in an unrelated control knockout, *Sc.bpt1Δ*. Thus, *Sc *but not *Cg *is aided by adding ergosterol to the environment, and this process is likely dependent on *AUS1 *and/or *PDR11*.

Three additional lines of evidence support the hypothesis that *Sc *prefers sterol import while *Cg *prefers fluconazole export in response to fluconazole treatment. A retrospective analysis of deletion mutant fitness in *Sc *[48] revealed that a greater proportion of gene deletions involved in the sterol pathway lead to fluconazole sensitivity than deletion of fluconazole transporters themselves (Fisher exact test, one-tailed *P = *0.043). This suggests a role for sterol transporters in the *Sc *fluconazole response. Second, fluconazole tolerance in *Cg *has been shown to be unaffected when constitutively expressing *CgAUS1 *in the presence of exogenous free cholesterol (though not in the presence of serum) [49]. Third, deletion of the *Cg *orthologs of fluconazole transporters *PDR5 *(*CgCDR1*) [50] or *SNQ2 *[51] both resulted in increased fluconazole sensitivity.

### Expression of sterol importers in *Kl *increases fluconazole tolerance

Since *Kl *neither up-regulates drug exporters nor encodes sterol importers, we considered that this lack of a transport response might be responsible for the higher drug sensitivity we observed for *Kl *in relation to the other species. Consistent with this hypothesis, we found that *Kl *growth was unaffected by addition of exogenous ergosterol (Figure 5c), similar to *Cg *but in sharp contrast to *Sc*. We also predicted that transgenic expression of sterol importers ScAus1 or ScPdr11 in *Kl *might increase fluconazole tolerance in the presence of exogenous ergosterol. To test this prediction, we chromosomally integrated *ScAUS1 *and *ScPDR11 *into *Kl *non-disruptively at the *KlLAC4 *locus under control of the strong constitutive *Kl *P_LAC4-PBI _promoter (Materials and methods). Transformed *Kl *strains were grown under fluconazole treatment with and without exogenous ergosterol (4 μg/ml). We observed that transgenic expression of sterol importer *AUS1 *in *Kl *significantly increased fluconazole tolerance (*P *= 0.012; Figure 5c) in an ergosterol-dependent manner. Thus, it appears that differences in sterol import and drug export are responsible for a component of the anti-fungal response, and of the observed functional divergence across the three yeast species.

## Conclusions

In this study, we have compared the dynamic transcriptional responses of three diverse yeast species to fluconazole treatment, revealing significant divergence in their regulatory programs. The data suggest several different mechanisms of azole tolerance, depending on the species (Figure 5d). The *Sc *response depends on sterol influx, through up-regulation of *PDR11 *and *AUS1*. In contrast, the *Cg *response relies on fluconazole efflux through strong up-regulation of *SNQ2 *and a *PDR5*/*10/15 *ortholog. Neither of these responses have evolved in *Kl*, leading to its severe drug sensitivity. These conclusions are supported by follow-up experiments demonstrating that growth in ergosterol increases the fluconazole tolerance of *Sc*, but not other species, in a *PDR11*- and *AUS1*-dependent fashion. They are also supported by the finding that transgenic expression of *AUS1 *in *Kl *increases the fluconazole tolerance of this species.

To arrive at these conclusions, we employed a novel 'soft clustering' approach that is of general use in the fields of comparative and systems biology. This approach is distinct from other methods for cross-species expression analysis [27,28,30,52] in several important ways. Chief among these, it integrates sequence orthology with gene expression patterns to produce accurate orthologous clusters. This integration is accomplished by a symmetric process that does not require the designation of one species as a reference. In addition, soft clustering handles data from more than two species and can, in principle, analyze any number of species simultaneously. In future, such approaches might be used to survey a wider range of species, drug concentrations and stimuli to reveal conserved and divergent molecular response pathways.

## Materials and methods

### Strains and growth conditions

Standard laboratory strains with known genomic sequence [53] were used: *Sc *BY4741, *Cg *CBS138 (ATCC 2001), and *Kl *NRRL Y-1140 (ATCC 8585). Cultures were grown in rich media (YPD) from OD_600 _of 0.05 to 0.2 at 30°C and 225 rpm. Cells were treated with fluconazole at species-specific sub-inhibitory concentrations (Figure S1 in Additional file 1), and harvested at 0, 1/3, 2/3, 1, 2 or 4 doubling times as measured for untreated cells.

### Microarray expression profiling

RNA was isolated by hot phenol/chloroform extraction and enriched for mRNA via poly-A selection (Ambion 1916, Austin, TX, USA). mRNA from untreated cells was combined in equal amounts from all time points to form a species-specific reference sample. Six replicates per time point were dUTP labeled (three biological replicates by two technical replicates) with Cy3 and Cy5 dyes (Invitrogen SKU11904-018, Carlsbad, CA, USA) creating a dye-swapped reference design. Samples were hybridized to Agilent expression arrays using the protocol recommended by Agilent. Differential expression was called using the VERA error model [54] and false discovery rate multiple-test correction [55]. Additional description of both the microarray platform and analysis can be found in Additional file 1.

### Soft clustering algorithm

We developed a constrained clustering method based on the *k*-means algorithm, but using a revised objective function (Additional file 1). Like regular *k*-means, the objective function considers the similarity of each gene's expression profile to the center of its assigned class. However, it also rewards class assignments in which orthologs are co-clustered. The reward (*W*) is a user-defined parameter that serves as a tradeoff between cluster expression coherence and percentage of co-clustered orthologs: each gene, *x *∈ *X*, is assigned to cluster h* such as to minimize the objective function:

$$h^{*}=\underset{h}{\arg\min}(\sum \left(D(x,C_{h})-W\right))$$

where ∑(D(*x*, *C_h_*) - *W*) refers to all possible partitions of genes in the same orthology group, *D() *refers to a user defined distance function, and *C_h _*denotes the center of cluster *h*. As discussed in the main text and in Additional file 1, the appropriate value of the reward, *W*, can be determined using complementary information. Here, it was tuned to maximize the GO enrichment of the clusters.

The new objective function also leads to changes in the search algorithm for determining the optimal cluster assignments: for each group of orthologs across the three species, we search for the partitions that result in the minimum total distance between all pairs of group members. Since there are 2*^m ^*possible subgroups, where *m *is the size of the orthology group (here, most orthology groups are of size *m *= 3), and each subgroup is checked for all possible *k *clusters, the search complexity for each group is O(2^m ^* *k*). Since *m *is small, the running time of the algorithm is typically very fast. Detailed methods, including algorithm pseudo-code, are presented in Additional file 1.

### Identifying highly conserved and divergent pathways

We first ranked GO processes categories [38] based on their significance of overlap with differentially expressed orthologous groups [32]. An orthologous group was considered differentially expressed if at least one member was differentially expressed. We used the top 20 ranked GO processes for identifying conserved and divergent pathways. Conserved pathways were defined as those with the highest 'full co-clustering' fraction of genes known to be involved in the process and divergent pathways were defined as those with the highest 'no co-clustering' fractions.

### Insertion of *ScAUS1*/*ScPDR11 *into *Kl *

To facilitate insertion of *ScAUS1 *and *ScPDR11 *into *Kl*, open reading frames were placed under control of the strong *P_LAC4-PBI _*promoter by cloning into plasmid pKLAC2 (NEB N3742S), which possesses approximately 2-kb homology to the *Kl.LAC4 *locus. Open reading frames were amplified with a *Sac*I restriction site (3' end), which was used to ligate a kanamycin marker from pCR-Blunt (Invitrogen K-2800-20). *Xho*I (5' end) and *Sbf*I (3' end) restriction sites were added by PCR for ligation into pKLAC2. Modified plasmids were transformed into *Escherichia coli *and screened on Luria-Bertani media containing ampicillin and kanamycin. Plasmids were mini-prepped (GE Healthcare #US79220-50RXNS, Piscataway, NJ, USA) and verified by PCR and *Sac*II digestion. All restriction enzymes were obtained from New England Biolabs (Ipswich, MA, USA).

*Sac*II-linearized plasmids were transformed into *Kl *NRRL Y-1140 by electroporation, thereby inserting *ScAUS1 *and *ScPDR11 *non-disruptively at the *Kl.LAC4 *locus. Colonies were selected on YCB + 5 mM acetamide (New England Biolabs N3742 S and verified by PCR. mRNA expression of *ScAUS1 *and *ScPDR11 *was validated by quantitative RT-PCR.

### Data

The data reported in this paper have been deposited in the Gene Expression Omnibus database, accession number [GEO:GSE15710].

## Abbreviations

ABC: ATP-binding cassette; *CG: Candida glabrata*; GO: Gene Ontology; *Kl: Kluyveromyces lactis*; MFS: major facilitator superfamily; *SC: Saccharomyces cerevisiae*.

## Competing interests

The authors declare that they have no competing interests.

## Authors' contributions

DK, KT, TR and TI designed the study. DK performed all experimental work. ZBJ and GZ developed the soft-constraint clustering approach. DK, KT, and GZ analyzed the data. DK and TI wrote the manuscript. ZBJ and TI supervised the work.

## Supplementary Material

Additional file 1 **Supplementary Methods, Results, and Discussion**. Click here for file

Additional file 2 **Supplementary Table S1**. Click here for file

Additional file 3 **Supplementary Table S2**. Click here for file
