# Supplementary material for: Evolutionary divergence in the fungal response to fluconazole revealed by soft clustering
Source: Genome Biol. 2010 Jul 23;11(7):R77. doi: 10.1186/gb-2010-11-7-r77 (PMC2926788; doi:10.1186/gb-2010-11-7-r77)
Supplement: Additional file 1 — Supplementary Methods, Results, and Discussion. [file gb-2010-11-7-r77-S1.DOC]

##### Supplementary Methods:

##### Selecting Yeast Species:

While previous comparative expression studies have focused on evolutionarily distant species such as *Candida albicans*, and *Schizosaccharomyces pombe*, we wished to compare multiple yeast species at a comparatively closer evolutionary distance. Therefore, we chose to study *Cg* since it is closely related to *Sc* yet remains phenotypically distinct residing well outside the *Saccharomyces sensu stricto* group. In choosing an appropriate species as an outgroup, we noted that another study [24] had shown that the whole genome duplication event ushered in large transcriptional and phenotypic changes among yeasts. Since both *Sc* and *Cg* had undergone this whole genome duplication, we chose *Kl* as an outgroup since the last common ancestor of these three yeasts dates to just before this event. In contrast, the last common ancestor of *Sc*, *Cg* and *Ca* is much more ancient [56].

In addition, *C. albicans* differs dramatically from *Sc* and *Cg* in at least two ways. Unlike other yeasts, *C. albicans* commonly undergoes phenotypic switching in which up to seven different cell types can be exhibited. While most of these phenotypes have not been extensively studied, large differences in transcriptional regulation between two of these morphologies have been shown (white and opaque switching). Therefore, it is unclear which phenotype is most appropriate for comparison with other yeasts.

*C. albicans* also exists as a diploid organism in contrast to *Cg* which exists uniquely as a haploid. While *Sc* and *Kl* can be isolated as either haploids or diploids, the vast majority of studies on both organisms have been performed on haploids. To further complicate matters, *C. albican*s possesses widespread and extensive heterozygosity among clinical isolates and even in comparison to other genomes. Thus comparison of haploid genomes such as *Sc*, *Cg* and *Kl* with that of the diploid *Candida albicans* genome would introduce unnecessary complications to our study.

##### Growth Conditions:

Optimal growth temperature for all three species in YPD media was determined to be 30^◦^C (Supplementary Figure 1A). Cultures were inoculated from a single colony and grown overnight. Cultures were then diluted to an OD_600_ of 0.1 and grown for two doublings. OD_600_ measurements were taken at 20 minute intervals for two more doublings. This data was then log2-transformed and fit with linear least square regression to determine doubling time. Three biological replicates were performed for each species. Doubling times were *Sc* BY4741 - 1.58 hrs, *Sc* YJM789 - 1.65 hrs, *Cg* - 1.1 hrs and *Kl* - 2.1 hrs.

##### Testing Fluconazole Susceptibility

Overnight cultures were diluted to OD_600_ of 0.001 in YPD media with varying amounts of ﬂuconazole and allowed to grow a time proportional to 11 log-phase doublings times. We determined 50% inhibitory concentrations of fluconazole to be: *Sc* BY4741 - 4µg/mL, *Sc* YJM789 – 0.25µg/mL, *Cg* - 4µg/mL, *Kl* – 0.0625µg/mL.

Fluconazole susceptibility in ergosterol-supplemented media was performed as previously described with synthetic complete (SC) media rather than YPD. Ergosterol was added to the media at 4µg/mL from a stock solution (8.75mg/mL ergosterol, Fluka # 45480 in 42% Tween-80 and 58% ethanol). Synthetic complete media lacking ergosterol with an equivalent amount of Tween-80 and ethanol was used as a control.

##### Microarray Design and Analysis:

For *S. cerevisiae*, samples were hybridized to the Agilent Yeast Microarray v2 (Catalog # G4140B). *C. glabrata* and *K. lactis* samples were hybridized to species-specific microarrays designed in-house. Oligonucelotide probe sets (60 mer) were designed to avoid: (1) tendency to self-dimerize (Mfold); (2) low-complexity sequences (RepeatMasker); (3) cross-hybridization to other genomic regions (WUBLAST2); (4) known repetitive sequence elements (RepeatMasker); (5) placement near the 5’ end of the gene (the 3’ end is preferred for maximum specificity); and (6) high and low melting temperatures very different from other oligos on the array (Mfold). Custom microarrays were manufactured using Agilent technology.

Custom microarray designs were validated by measuring the mRNA expression of several genes for both *C. glabrata* and *K. lactis* using RT-qPCR (Supplementary Figure 2). Six genes were chosen per species (two up-, two down-regulated and two non-changing and compared against a housekeeping gene - *CgACT1* or *KlACT1*) for validation.

Arrays were scanned using a GenePix 4000A microarray scanner and quantified with GenePix 6.0 software package. Data from each array was subsequently subjected to background subtraction and LOESS normalization with spatial correction. The intensity values of within-array technical replicates (identical probes on the same array) were averaged. Missing values in the data set were imputed using the KNNImpute algorithm [57]. Quantile normalization was applied separately to the data for each species.

We compared the *Sc* differentially expressed genes with single gene deletions conferring fluconazole tolerance or sensitivity [48]. Supplementary Figure 5 shows the number of common genes between the two studies at various thresholds (when taking the top genes). Similarly to previous studies comparing MMS deletion fitness profiling and gene expression [58, 59], we found that the transcriptional response to fluconazole treatment did not correspond to gene deletions which protect against fluconazole. However, among the top gene deletions which conferred sensitivity to fluconazole included several genes of the ergosterol biosynthetic pathway (*ERG11*, *ERG8* and *ERG13*).

We also compared the *Sc* differentially expressed genes with those considered differentially expressed in previous studies [17, 60]. Since only total numbers of differentially expressed genes and not *p*-values of differential expression were previously reported, we non-parametrically compared our list of differentially expressed genes with literature by considering only the same number of genes between studies (e.g. only the top 116 and 62 top differentially expressed genes, respectively). Despite such studies being performed on different strain isolates, at different drug concentrations, at different treatment times, and sometimes with slightly different azole drugs, we found significant overlap between our data and previously published data (Supplementary Figure 5).

Next we compared our data with a previous study on *C. albicans* [13]. As before, we compared our list of differentially expressed genes by considering the same number of genes between studies (507 genes). While many of the same GO categories were enriched between studies (e.g. ergosterol biosynthesis, and oxido-reductase activity), we found only weak enrichment for the overlap in differentially expressed genes between *Sc* and *Ca* and no significant enrichment with *Cg* or *Kl* (Supplementary Figure 6).

##### Constrained Clustering Algorithm:

A major challenge in clustering gene expressions data is that cluster membership is often influenced by small changes which can be attributed to noise (especially when many clusters are used which is often the case when thousands of genes are analyzed together [61, 62]). When clustering genes from multiple species, this noise can lead to orthologs with similar expression profiles being assigned to different clusters. To overcome this, we developed a soft constraint [33, 63, 64] clustering framework based on *k-*means clustering which incorporates other data sources influencing the resultant clusters. For clustering data from multiple species we use orthology relationships as a constraint.

The term “soft clustering” has also previously been used in other clustering methods to define cases in which a gene can belong to more than one cluster rather than any constraint used to identify clusters [65, 66]. For our method, “soft clustering” refers to the prior we use as a weight to encourage co-clustering of orthologous genes. In these cases, “soft” refers to the assignment of genes to clusters.

Other methods which focus on the analysis of expression levels across species are limited to simultaneous analysis of two species or require assumptions regarding the distribution of expression data [27-29].

##### Algorithm Pseudocode and Implementation:

SoftClust(data set *X*, # of clusters *k*, distance metric *D*, orthology relations *Rel_Orth_*, orthology weight *W_orth_*)

1. Let *C_1_…C_k_* be the *k* initial cluster centers.
2. Each gene $x\in X$ is assigned to cluster *h** (i.e., to set $X_{h*}^{(t+1)}$) for:

where$\sum_{I(x,x_{i},x_{j})\in\mathrm{Re}l_{Orth}} (D(x,C_{h}^{(t)})-W_{orth})$ refers to all possible partitions of genes in the same orthology group into clusters based on the distance metric, *D*, by calling the recursive function: *PartitionSet(G)*.

1. For each cluster *h*, update its center by averaging all gene profiles assigned to it in step 2.
2. Iterate between (2) and (3) until convergence.
3. Return {*C_1_…C_k_*}.

PartitionSet(orthology set *G*)

1. If |*G*| is 1, calculate distance of the gene to all clusters. Store the distance value and the best assignment.
2. Otherwise, for each possible partitioning (*P^i^*) of *G* to *j* sub groups, *j*>1
   1. For each sub group *g^j^* in *P^i^*
      1. If optimal partitioning of *g^j^* was already calculated, use the stored partitioning and distance value.
      2. Otherwise PartitionSet (*g^j^*).
   2. *Distance value of P^i^* = sum of optimal distance values for all sub groups g*^j^*.
   3. If the *Distance value of P^i^* is minimal, keep distance value and partitioning.
3. Calculate the reward for clustering *G* together. Find the cluster that minimizes the rewarded assignment of *G* when all genes are clustered together.
4. Store the minimal distance value (step 2 & 3) for corresponding partitioning of *G*

This algorithm was implemented in the Java programming language and will be made available upon request.

##### Selecting Parameters for the Constrained Clustering Method:

Similar to the standard *k*-means clustering we need to specify the number of clusters (*k*). In addition we need to choose an appropriate reward weight (*W_orth_*). We tested various number of clusters and reward weights using the total number of enriched GO terms (Bonferonni-corrected *p* ≤ 0.05) as an external objective measure for selecting the values of these parameters. Figure 2C shows the median number of enriched GO terms for 50 runs at different *k* and *W_orth_*. A clear enrichment of GO terms is seen for reward weights between 0.75 and 1.5. We chose the conservative parameters of *k =* 17 and *W_orth_ =* 0.75. While *W_orth_* = 1.0 obtains a higher number of enriched GO terms than our choice of *W_orth_ =* 0.75, the associated variance from *W* = 0.75 to 1.0 increases by approximately 1.2% whereas the increase in variance from *W* = 0 to 0.75 is twenty fold smaller (0.06%). As we already observe large increases in ortholog co-clustering with a miniscule increase in cluster variance from *W* = 0 to 0.75, we believe that the more conservative choice of *W* = 0.75 is appropriate.

In order to examine the effect of the reward on cluster size, we plotted cluster sizes for a range of values. For each value, 50 runs were tested. Results for clustering experiments with *k*=10,15,20,25 are summarized in Supplementary Figure 4B. These results demonstrate that cluster size does not significantly change with various reward values.

##### Constrained Clustering Leads to only a Small Increase in Inter-Class Variance:

Our clustering method imposes a delicate balance between achieving noise reduction, more biologically meaningful clusters, and forcing divergent expression profiles to co-cluster. We assessed this balance by measuring the increase of the within-cluster-variance between rewarded and standard *k*-means runs. Increasing the reward weight results in increased cluster variance which are affected by orthology relationships. We computed the cumulative cluster variance for various reward weights. As can be seen (Figure 2D), the variance only marginally changes when using the reward selected for this set (*W_orth_ =* 0.75). A marked increase in the variance is seen for larger reward weights. This indicates that at the selected reward level expression profiles still plays a major role in cluster assignments. The reward (which is based on orthology relationships) is used only to move genes to clusters that provide a good fit in terms of expression profiles (even if not optimal). Thus, the reward achieves its intended goal: Identifying co-clustered orthologs while not dramatically affecting the resulting cluster profiles.

##### Randomizing Orthology Assignments Significantly Decreases Co-clustered Orthologs:

The underling assumption when using the orthology information to aid the clustering of gene expression, is that we expect orthologs to have similar expression profiles. To test this assumption we randomized the orthology mappings by assigning genes in one species to random genes in another while keeping the same number of orthology relationships. Next we applied our soft constraints clustering method using the randomized mapping. As can be seen (Figure 2D, top graph) in the randomized mapping, the number of enriched GO terms is markedly reduced and the variance of the randomized mapping is larger for our selected reward value of *W_orth_ =* 0.75. The increase in variance is a direct result of the decrease in expression similarity between genes considered orthologs. This indicates that the true orthology relationships are indeed between similarly expressed genes. Moreover, the fraction of co-clustered orthologs is about 50% lower for the randomized mapping when using *W_orth_ =* 0.75 since this reward is not large enough to encourage distinct expression profiles to cluster together (Figure 2D, bottom graph). Combined, these results support our assumptions regarding the similarity in expression of orthologous genes.

Lastly, as the *k*-means algorithm is initialized with random selection of the centroids, different clusters are produced for each run. We ran the clustering algorithm with the selected parameters for 50 times, noted the fraction that each two genes were clustered together out of the 50 runs, gathered the co-clustered genes in a matrix, and applied hierarchical clustering to the co-clustering matrix. A clear clustering structure is revealed (Figure 2E).

##### Randomizing the Orthology Mapping:

Two randomizations were employed; ortholog randomization and paralog randomization. For ortholog randomization, each gene was randomly substituted with a different gene from the same species, thus keeping orthology relationships intact. For paralog randomization, one of the paralogs was selected to be part of the orthology set. The remaining paralogs were randomized while keeping the true orthology relationships intact. It should be noted that only 443 orthology groups contained paralogs out of 4275 ortholog sets (10.3%).

##### Co-clustering Matrix:

The fraction of times each pair of genes were clustered together in 50 runs of the algorithm was noted for all possible gene pairs, thus creating a similarity matrix for all genes. Hierarchical clustering was applied on the similarity matrix using average-linkage clustering. The result is shown in Figure 2E.

##### Analysis of Doubling Time Points vs. Absolute Time Points:

We assessed the impact of using the number of doubling times in lieu of absolute times when choosing points for the time course. Since *Cg* had the shortest doubling time, we linearly interpolated the time courses of *Sc* and *Kl* to match that of *Cg*. We re-ran our clustering algorithm with the same parameters (*k* = 17, *W* = 0.75) and compared the number of co-clustered orthologs between species (Supplementary Figure 3). We found that using doubling time greatly increased the number of co-clustered orthologs as compared to using absolute (interpolated) time points.

##### Motif Analysis:

Four DNA-motif ﬁnding methods were used on each cluster: AlignACE [67], MEME [68], Weeder [69], and Consensus [70]. Default parameters were used for each method. The resulting position weight matrices (PWMs) from each method were used to scan species-specific promoter regions (using Patser [70]) for enrichment via the hypergeometric test. A promoter region was considered bound for MotifScore ≥ 0.7. MotifScore is calculated as the fraction of the maximum possible information content for the motif. All species intergenic regions were used as background for calculation of information content. Enriched PWMs were compared to known *S. cerevisiae* PWMs [71, 72] using the STAMP software package [73].

As an alternative to *de novo* DNA motif finding, we also used pre-defined PWMs [71, 72] to directly scan promoter regions using Patser [70]. Promoters were considered bound as previously described. This approach permits the calculation of an enrichment score for each PWM using the hypergeometric enrichment test followed by multiple test correction [55].

##### Expression Conservation of the General Stress Response:

To examine the evolution of the transcriptional regulatory mechanisms of the conserved stress-response genes, we used the technique of phylogenetic profiling. 19 fungal genomes were selected from the Fungal Orthgroups Repository [56]. Nine orthologous differentially expressed genes that possess both RRPE and PAC motifs in *S. cerevisiae, C. glabrata,* and *K. lactis* were used as a reference for the entire orthogroup. The PAC and RRPE PWMs were searched against the orthogroup fungal sequences using Patser [70], and sequences in which the motif was found with MotifScore > 0.7 were determined as containing the motif (Supplementary Figure 8). Previous work showed the PAC motif emerged during the *S. cerevisiae* - *C. albicans* divergence [31]. In contrast, our analysis suggests that the PAC motif first emerged by *Y. lipolytica* lineage and became well established in the fungal phylogeny by *A. gossypii*.

##### Species-specific Motifs:

Scanning the promoter regions of *Cg* and *Kl* genes predisposes us to elucidating regulation by transcriptional regulators with close orthology to their *Sc* counterparts. In order to identify putative transcription factor binding sites (TFBS) unique to *C. glabrata* and *K. lactis* (either novel TFs or TFs with highly diverged DNA binding domains) we performed *de novo* motif search on each of the clusters previously described. In addition, we verified that each discovered motif was species-unique by calculating its hypergeometric enrichment in both co-clustered genes and orthologs. We discovered two *Kl* TFBS in clusters 15 and 8 (Supplementary Figure 9, *p =* 6.51 × 10^-4^ and *p =* 5.80 × 10^-19^) showing high similarity (E ≈ 0) and a lack of enrichment in *Sc* and *Cg* clusters (*p* ≥ 0.109). *Kl* genes in clusters 15 and 8 possessing this putative motif lack *Sc* and *Cg* orthologs. These promoters are also enriched for the ScHac1p TFBS (*Sc*: *p =* 1.0, *Cg*: *p =* 0.158, *Kl*: *p =* 7.71 × 10^-7^). A search of known TFBSs from TRANSFAC [72] shows the *Kl* motif has high similarity to the MEF-3 TFBS in mouse (Supplementary Figure 9).

##### Transport:

*GCN20*, *NEW1* and *RLI1* are all strongly down-regulated in *Kl* while *CgNEW1* shows up-regulation and *Sc* orthologs show no changes in mRNA expression. These three genes are involved in nucleoside-triphosphatase activity (*p =* 7.03 × 10^-6^) in *Sc*. Scanning the orthologous promoters of these genes with known DNA binding motifs indicates that the difference in *Kl* regulation of these genes is linked to the Xbp1 DNA binding motif. We find all three promoters of the *Kl* orthologs possess Xbp1p TFBSs, while none exist in *Sc* or *Cg* orthologous promoters. In addition, *Sc*, *Cg* and *Kl* *XBP1* orthologs are all strongly down-regulated.

##### Analysis of Sterol Import Machinery in Fungal Genomes:

Since sterol synthesis requires oxygen [33, 47, 74], the emergence of sterol importers following the yeast whole genome duplication event was likely a key event in enabling *Sc* and *Cg* to grow anaerobically [75].

To examine sterol transport genes between species we used the Inparanoid algorithm [32] to search for potential orthologs of *AUS1* and *PDR11* in several pre- and post-whole genome duplication yeasts [56, 76]. We did not find orthologs of known sterol transporters in pre-whole genome duplication yeasts. These results likely indicate that *AUS1* and *PDR11* emerged after the whole genome duplication event (Supplementary Figure 10).

**Data deposition**: The data reported in this paper have been deposited in the Gene Expression Omnibus (GEO) database, www.ncbi.nlm.nih.gov/geo (accession no.GSE15710).

**Supplementary Figures:**


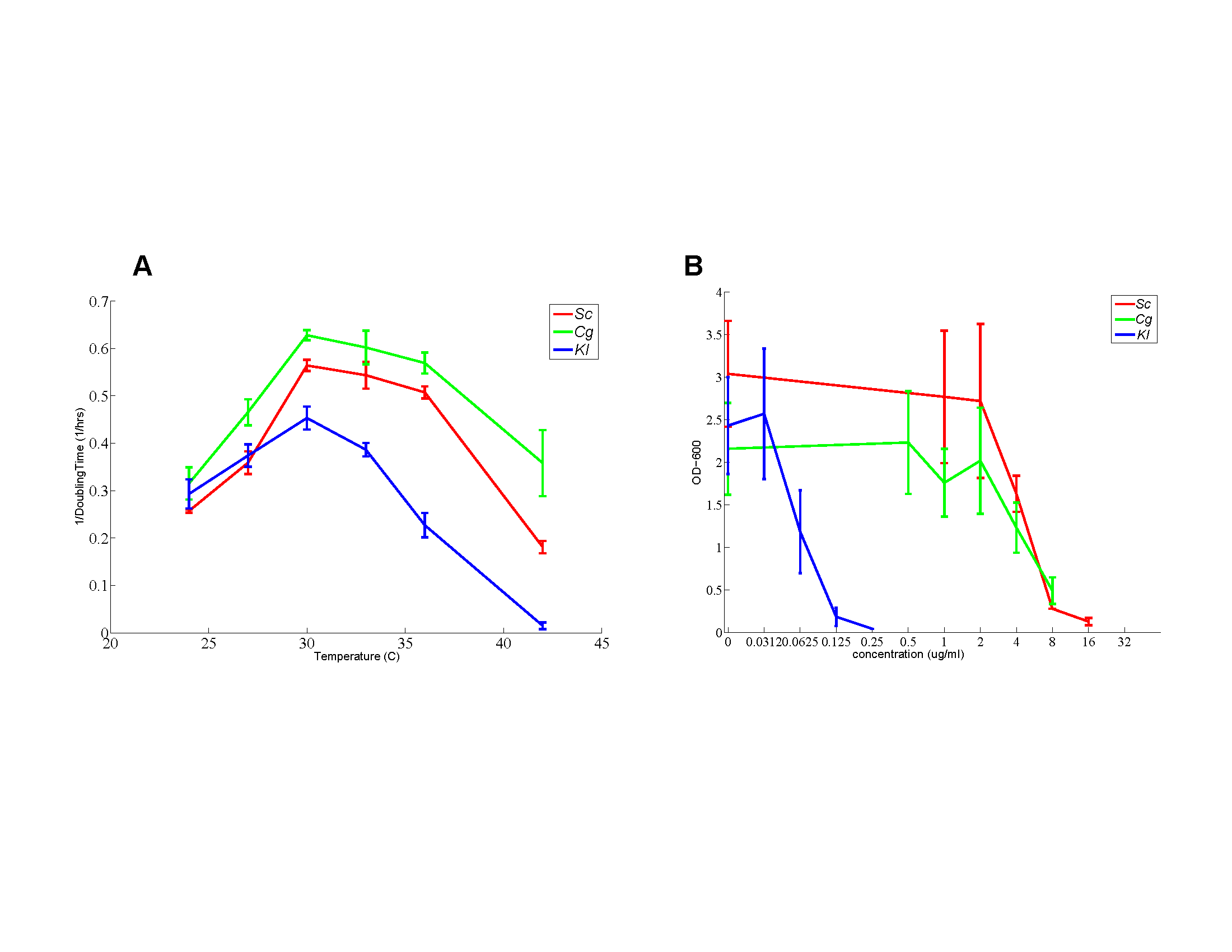


Figure S1. A) Doubling times during log phase growth for three biological replicates of the three species at different incubation temperatures. Overnight cultures were diluted to an OD_600_ of 0.1 and allowed to grow for two doubling log-phase doubling times. After this initial growth, OD_600_ measurements were taken at 30 minute intervals for two more doublings. The log_2_-transformed data was ﬁt with linear least-squares regression. Optimal growth occurred at 30^◦^C for all species. B) Growth curves for each species at different drug concentrations. Overnight cultures were diluted to OD_600_=0.001 and allowed to grow a time proportional to 11 log-phase doublings at various concentrations of ﬂuconazole.


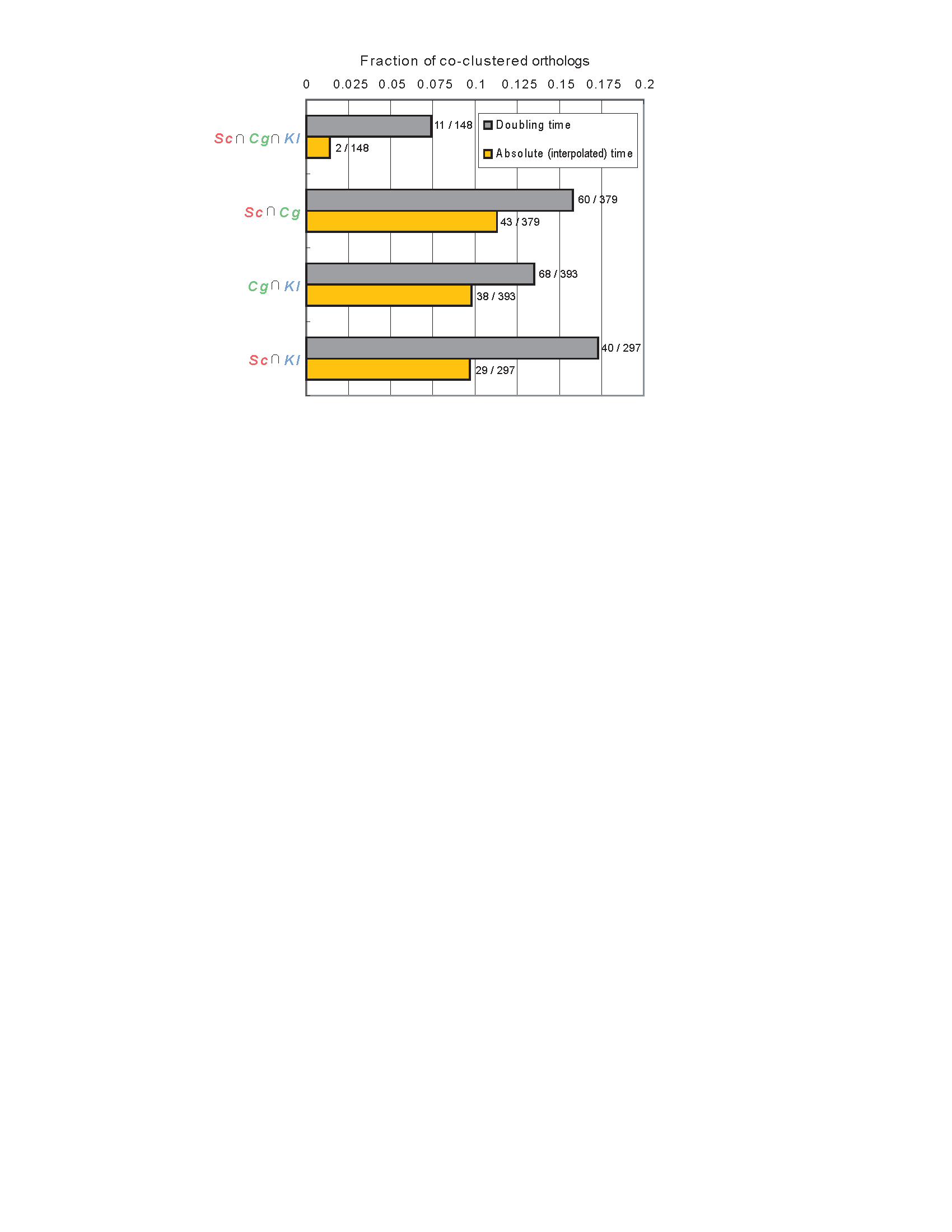


Figure S2. Analysis of the fraction of co-clustered orthologs using doubling time vs. absolute interpolated time. We find larger agreement between species when using doubling time.


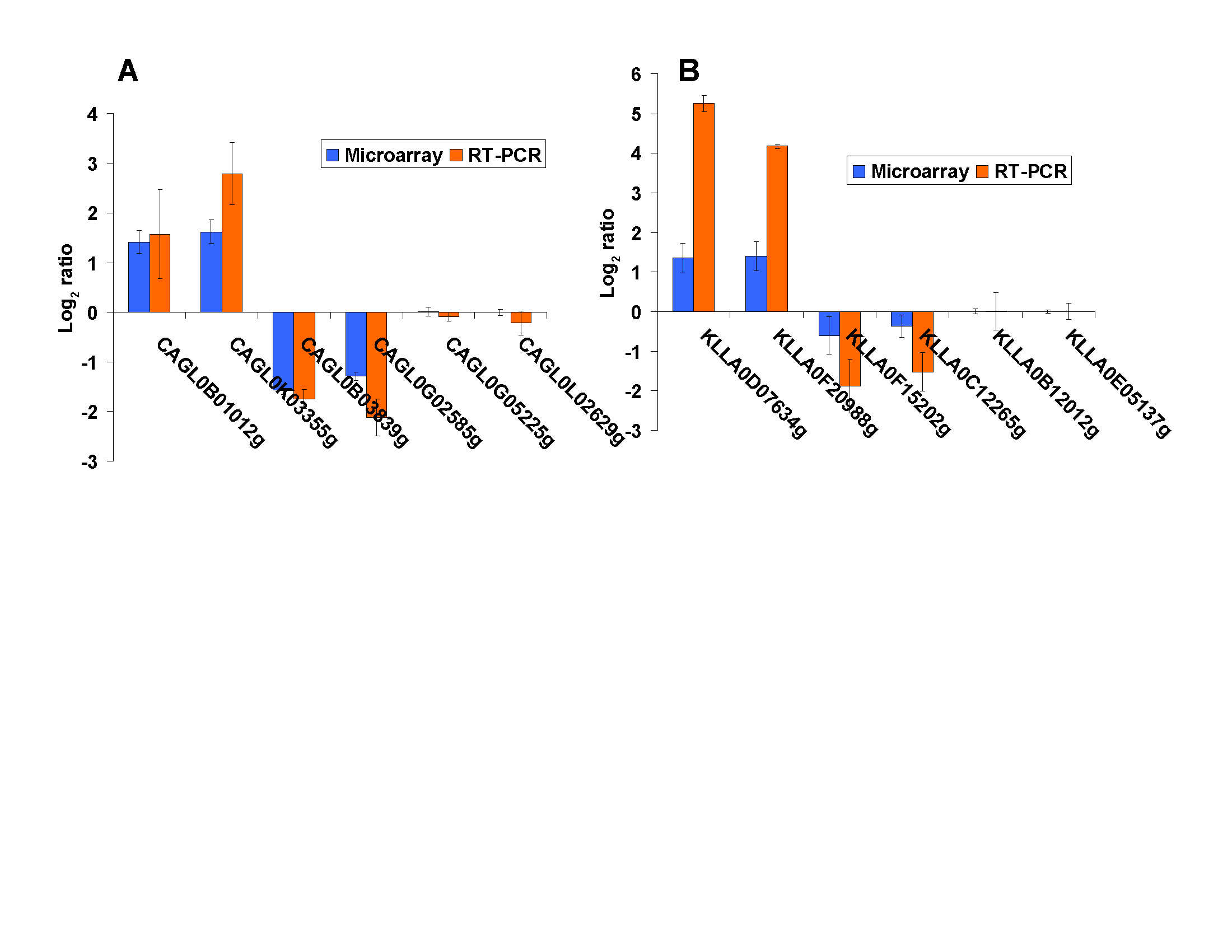
 Figure S3. RT-qPCR verification was performed for six *C. glabrata* and six *K. lactis* genes (two upregulated, two down-regulated and two unchanging) confirming our microarray results.


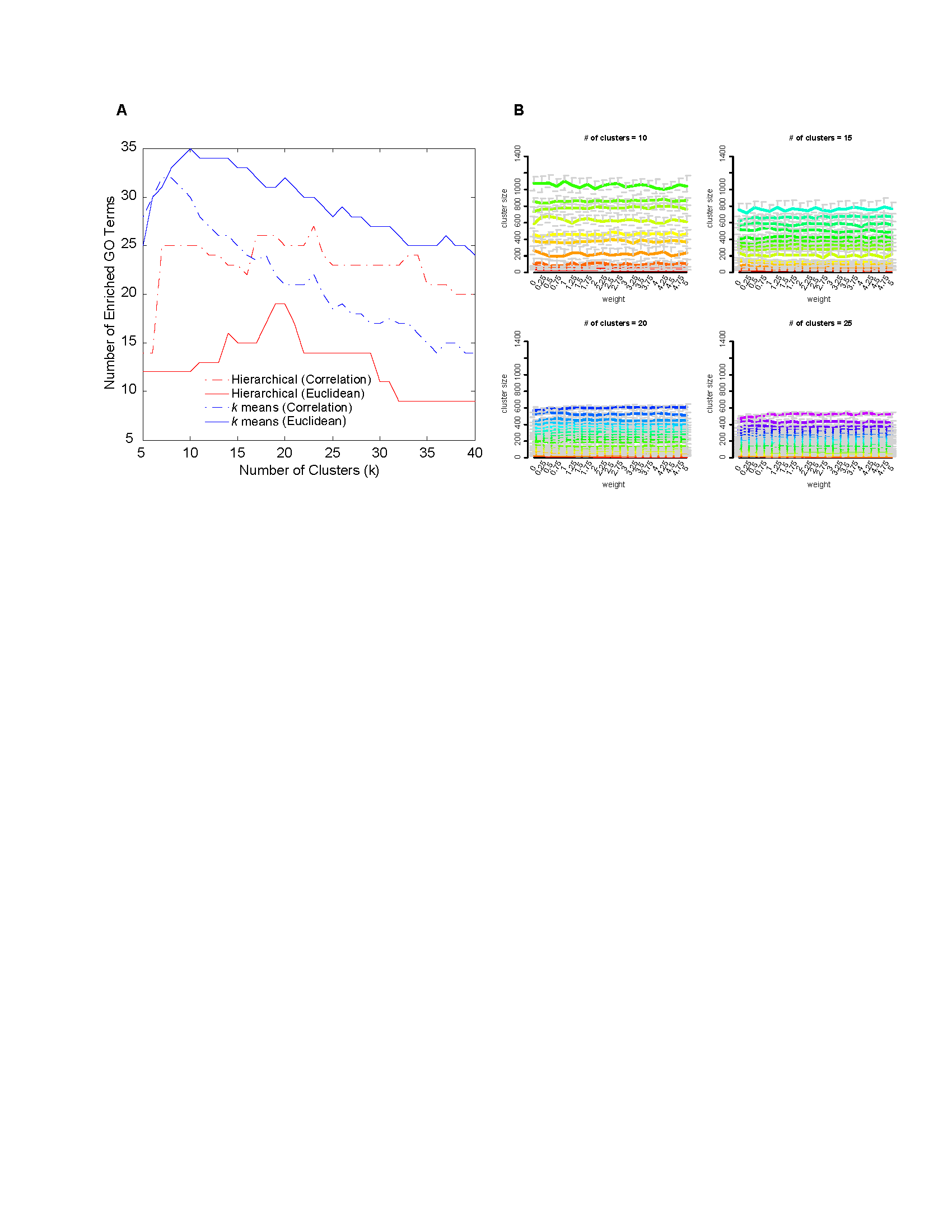


Figure S4. A) Comparison of the number of enriched GO terms using standard clustering algorithms (*k*-means and hierarchical clustering) and distance metrics (Euclidean distance and Pearson correlation). *k*-means clustering was performed 50 times for each parameter choice and the median GO enrichment is reported. B) Median cluster size (y-axis) over 50 runs for each of the n^th^ clusters (sorted by size) for various weights and values of *k*=10,15,20,25 clusters over 50 runs. Whiskers denote the cluster size upper and lower quartiles.


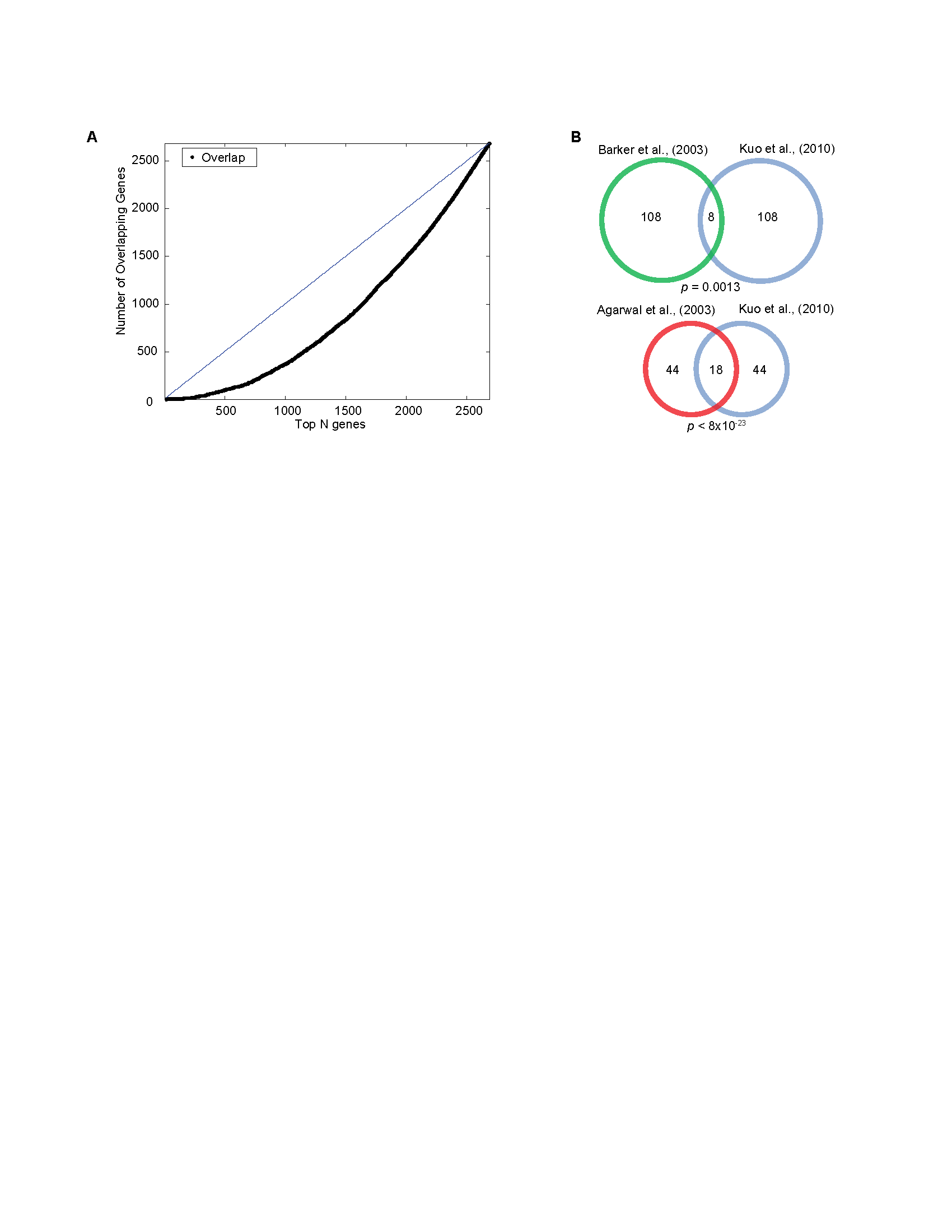


Figure S5. A) Comparison of top differentially expressed *Sc* genes with the top gene deletions conferring fluconazole sensitivity / tolerance [48]. B) The differentially expressed genes in our study significantly overlapped with previously published fluconazole microarray studies [17, 60].


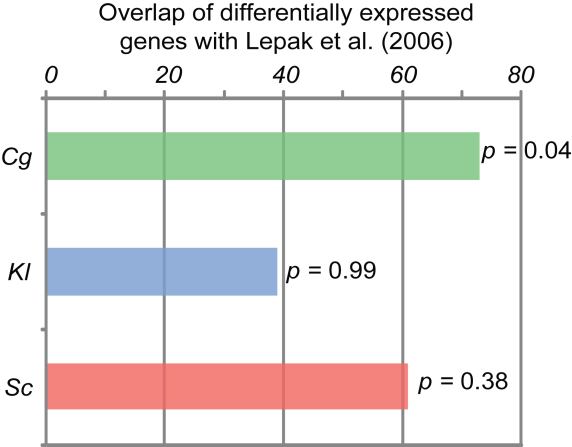


Figure S6. Comparison of data in this study with that of Lepak et al. [13].


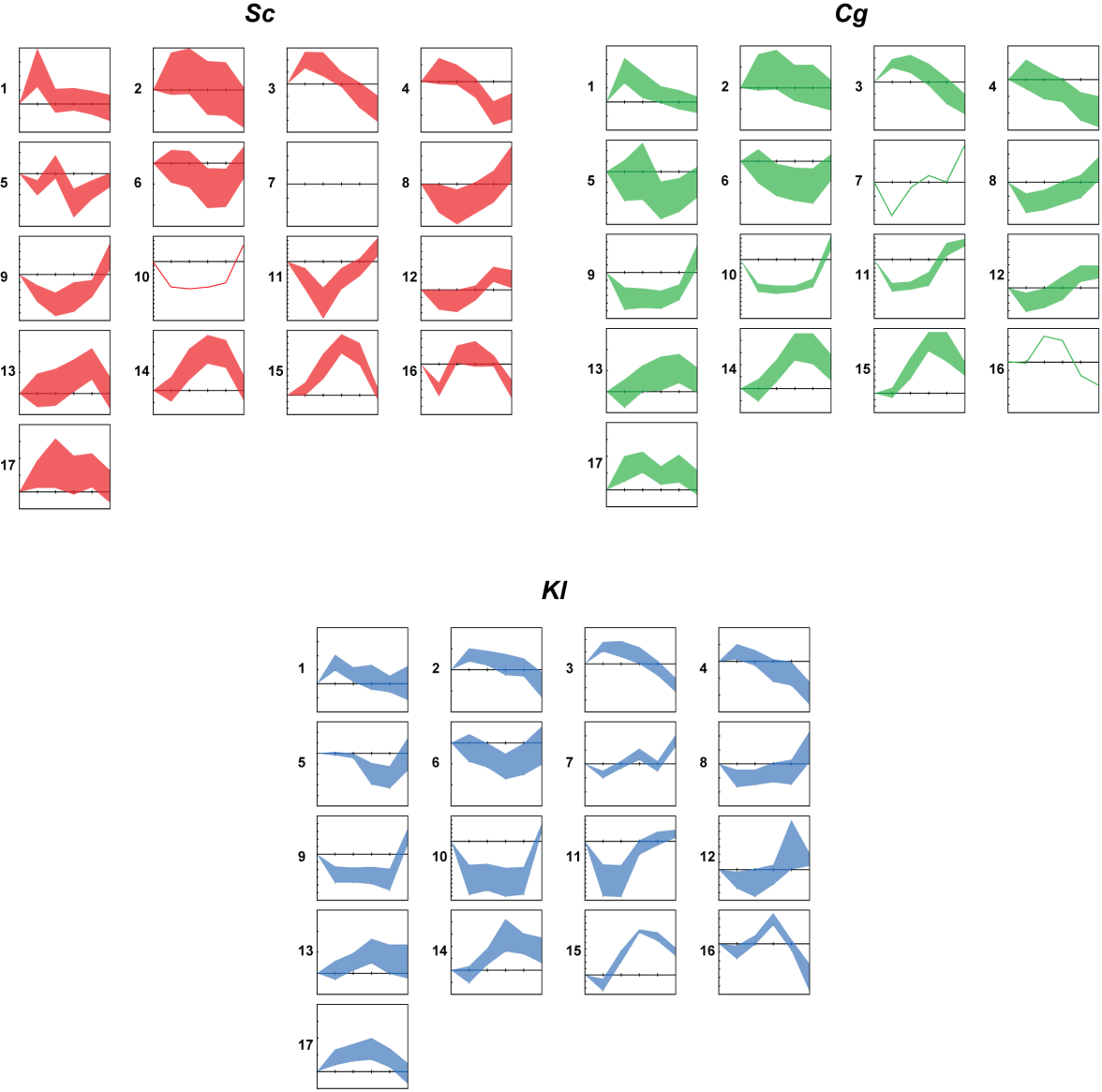


Figure S7. Plots of cluster dynamics for individual species. *Sc* – red, *Cg* – green, *Kl* – blue.


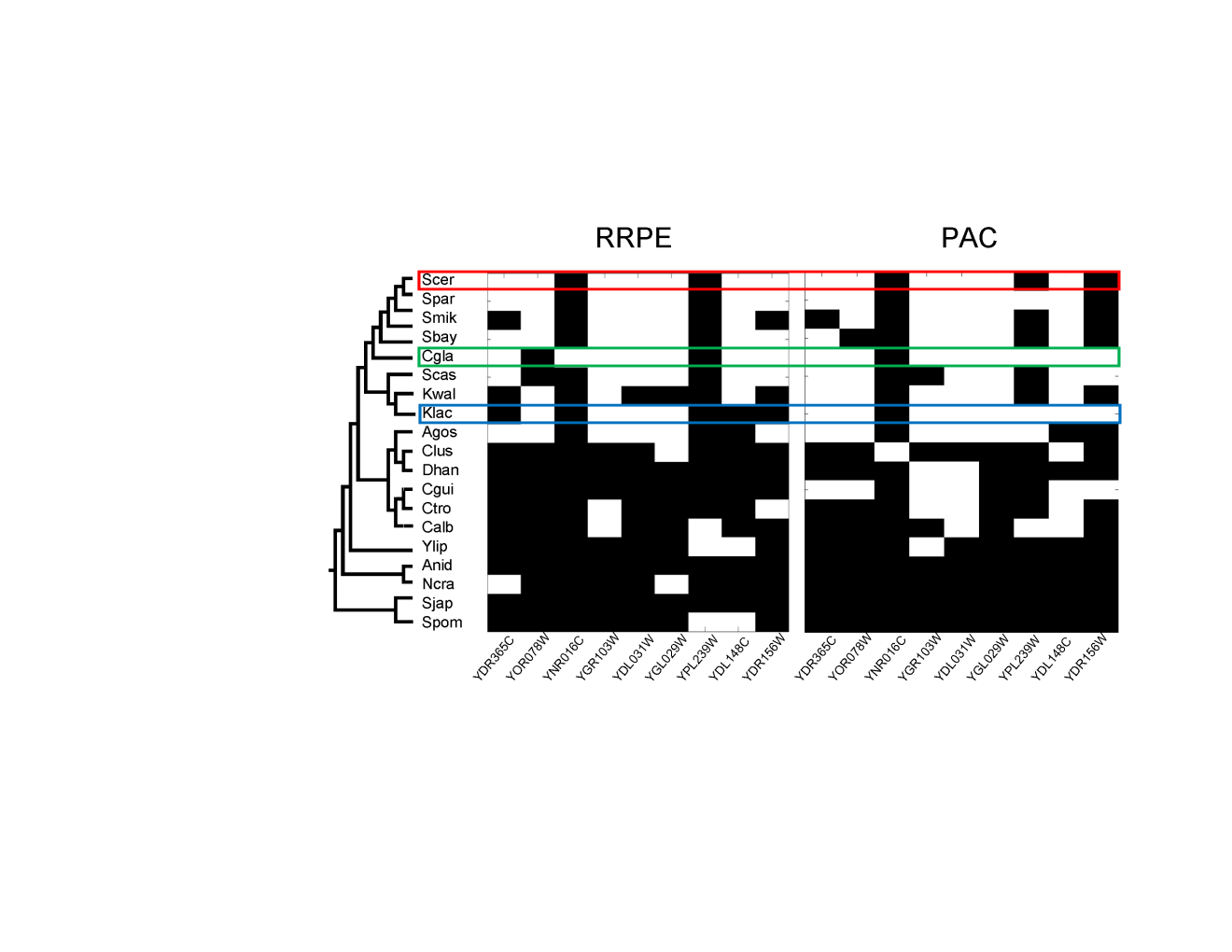


Figure S8. Phylogenetic profiling of PAC and RRPE motifs shows that these motifs become firmly established by the *S. cerevisiae* - *A. gossypii* lineage. *S. cerevisiae* gene names are used to denote the different yeast species orthologs. The presence / absence of motifs was determined using Patser.


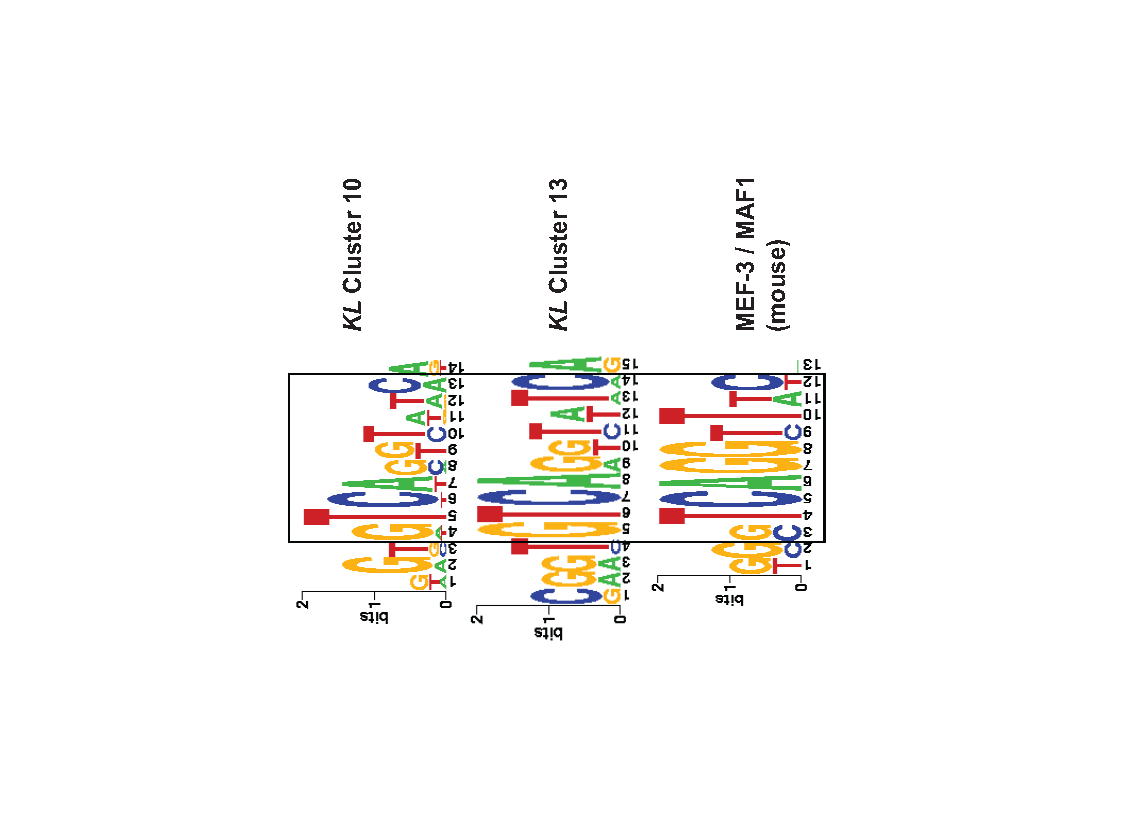


Figure S9. Species specific motifs. A *Kl* motif found in two clusters bears striking similarity to the Mef-3 mouse binding site.


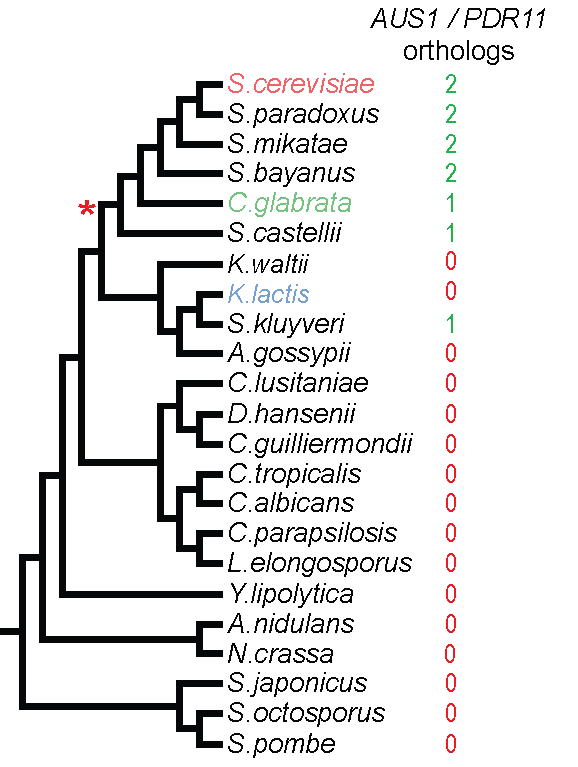


Figure S10. Inparanoid search for orthologs of *AUS1* and *PDR11* in fungal genomes reveals that these sterol transporters likely emerged after the whole genome duplication event.


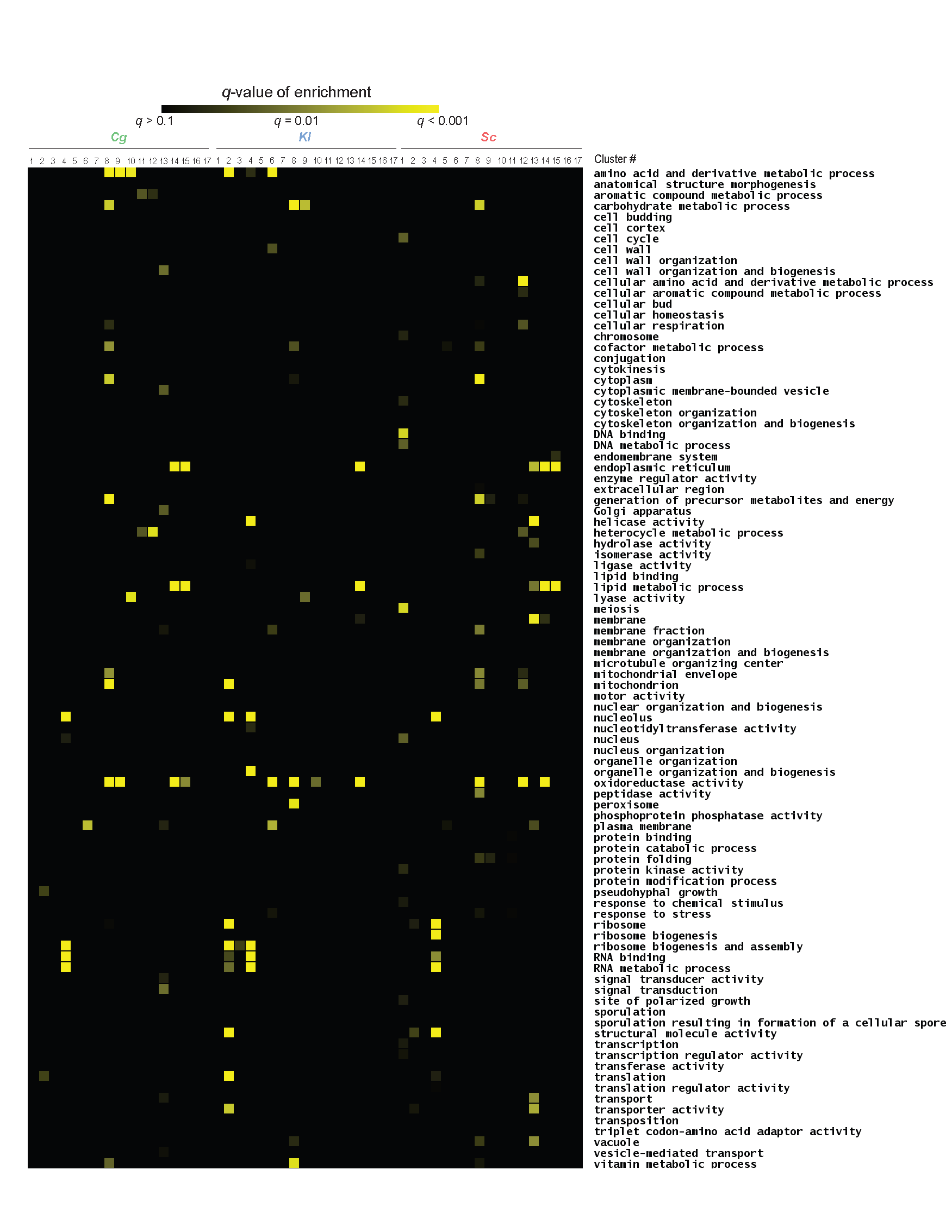


Figure S11. Full GO enrichment results for *Cg*, *Kl* and *Sc* clusters.

##### Supplementary Table Captions:

Table S1: Assignment of genes to clusters as determined by the soft clustering algorithm.

Table S2: Complete listing of co-clustering statistics for all GO categories. For each category the following are noted: the number of differentially genes in the category, the percentage of genes for which all three species were co-clustered, two species were co-clustered, no orthologs were co-clustered, no orthologs were differentially expressed, the number of differentially expressed orthologs in the category, and the total number of orthologs in the category. The top 20 most enriched categories are highlighted.
